# A data assimilation framework that uses the Kullback-Leibler divergence

**DOI:** 10.1371/journal.pone.0256584

**Published:** 2021-08-26

**Authors:** Sam Pimentel, Youssef Qranfal

**Affiliations:** 1 Department of Mathematical Science, Trinity Western University, Langley, BC, Canada; 2 Department of Mathematics, Simon Fraser University, Burnaby, BC, Canada; 3 School of Computing and Data Science, Wentworth Institute of Technology, Boston, MA, United States of America; King Abdullah University of Science and Technology, SAUDI ARABIA

## Abstract

The process of integrating observations into a numerical model of an evolving dynamical system, known as data assimilation, has become an essential tool in computational science. These methods, however, are computationally expensive as they typically involve large matrix multiplication and inversion. Furthermore, it is challenging to incorporate a constraint into the procedure, such as requiring a positive state vector. Here we introduce an entirely new approach to data assimilation, one that satisfies an information measure and uses the unnormalized Kullback-Leibler divergence, rather than the standard choice of Euclidean distance. Two sequential data assimilation algorithms are presented within this framework and are demonstrated numerically. These new methods are solved iteratively and do not require an adjoint. We find them to be computationally more efficient than Optimal Interpolation (3D-Var solution) and the Kalman filter whilst maintaining similar accuracy. Furthermore, these Kullback-Leibler data assimilation (KL-DA) methods naturally embed constraints, unlike Kalman filter approaches. They are ideally suited to systems that require positive valued solutions as the KL-DA guarantees this without need of transformations, projections, or any additional steps. This Kullback-Leibler framework presents an interesting new direction of development in data assimilation theory. The new techniques introduced here could be developed further and may hold potential for applications in the many disciplines that utilize data assimilation, especially where there is a need to evolve variables of large-scale systems that must obey physical constraints.

## Introduction

Data assimilation is the process by which we merge two types of information about a dynamic system, a numerical model of the underlying processes and observations of the evolving system. The resulting analysis should ideally be optimal in the sense of utilizing associated error and representativeness of the model and observations. The data assimilation procedure can be used to improve initial conditions, boundary conditions and/or parameter values of the numerical model, resulting in better estimates of the state of the system and improving predicability. Data assimilation is most prominently used in the atmospheric and oceanographic sciences where it is essential for modern numerical weather prediction [1]. In addition to being extensively used throughout the geosciences [2] it is increasingly being found a useful computational tool in a wide array of other disciplines, including medicine [3], epidemiology [4], ecology [5], and neurobiology [6].

In recent years there has been a renewed interest in the mathematical foundations of data assimilation (e.g., [7, 8]). One of the research developments being pursued is the consideration of different metrics for the model–observation differences and regularizer that are minimized in data assimilation algorithms. The standard data assimilation approach involves minimizing an objective function of weighted L_2_-norms, otherwise known as Tikhonov regularization. However, minimization involving a L_1_-norm for the regularization (or background) term has also been used and this is found to be particularly useful for tracking sharp fronts and discontinuities (e.g., [9, 10]). Rao et al. [11] found L_1_-norm data assimilation to be beneficial when dealing with outlier observations, but had the drawback that solutions lacked smoothness near the mean, this desirable property was retained by using the Huber-norm, a hybrid that utilizes L_1_ in the presence of outliers and L_2_ close to the mean. An alternative approach to data assimilation, explored by Feyeux et al. [12], utilizes optimal transport theory and within this context the Wasserstein distance (minimizing kinetic energy) replaces the L_2_ distance. Feyeux et al. [12] demonstrated how this approach holds potential for addressing position errors, see also Li et al. [13]. In this paper we introduce another alternative approach to data assimilation, one which explores an information perspective and uses the Kullback-Leibler divergence.

The Kullback-Leibler divergence (KL) between two probability distributions, *P* and *Q*, originally proposed by Kullback and Leibler [14], is defined as the expectation of the logarithmic difference between the probabilities *P* and *Q*, where the expectation is taken using *P*,

$$\begin{array}{c} \mathrm{KL}\left(P,Q\right)=E[\text{ln}(\frac{P}{Q})]. \end{array}$$ (1)

For two probability densities *P* and *Q* of a continuous random variable *x* we have

$$\begin{array}{c} \mathrm{KL}\left(P,Q\right)=\int_{-\infty }^{\infty }[P\left(x\right)\text{ln}(\frac{P(x)}{Q(x)})]\;dx \end{array}$$ (2)

and for two probability distributions of a discrete random variable

$$\begin{array}{c} \mathrm{KL}\left(P,Q\right)=\sum_{x\in X}P\left(x\right)\text{ln}\frac{P(x)}{Q(x)}. \end{array}$$ (3)

Sometimes referred to as the cross-entropy distance or relative entropy, the Kullback-Leibler divergence can be thought of as measuring the discrepancy between probability distributions, in this case the divergence of *P* from *Q*. From a Bayesian perspective it is a measure of information gained when an a priori probability distribution, *Q*, is updated to the posterior probability distribution, *P*. The Kullback-Leibler divergence is not strictly a true metric, and is not symmetric, hence in general KL(*P*, *Q*) ≠ KL(*Q*, *P*). This is because it is an expected value (Eq 1) and therefore it can differ depending on which distribution you take the expectation with respect to. Therefore, the Kullback-Leibler divergence is not a measure of distance in the usual sense but rather can be thought of as a directed, or orientated, distance; although a symmetric KL-functional, known as the Jensen-Shannon divergence (JSD), can be constructed as

$$\begin{array}{c} \mathrm{JSD}\left(P,Q\right)=\frac{1}{2}\mathrm{KL}\left(P,M\right)+\frac{1}{2}\mathrm{KL}\left(Q,M\right), \end{array}$$ (4)

where $M=\frac{1}{2}\left(P+Q\right)$.

A broader class of f-divergence was introduced by Csiszár (see, for example, [15]):

$$\begin{array}{c} D_{f}\left(P,Q\right)=\sum_{i=1}^{n}q_{i}f(\frac{p_{i}}{q_{i}}), \end{array}$$ (5)

where *f* is a convex function on (0, ∞) with *f*(1) = 0, *P* = (*p*_1_, …, *p*_*n*_)^⊤^ and *Q* = (*q*_1_, …, *q*_*n*_)^⊤^. Following Csiszar [15] the KL-divergence for arbitrary $P,Q\in R_{+}^{n}$ can be considered the f-divergence with

*f*(*t*) = *t* ln(*t*) − *t* + 1, that is

$$\begin{array}{c} \mathrm{KL}\left(P,Q\right)=\sum_{i=1}^{n}p_{i}\text{ln}(\frac{p_{i}}{q_{i}})+q_{i}-p_{i}. \end{array}$$ (6)

This is sometimes referred to as the generalized KL-divergence or the unnormalized KL-divergence; noting that if *P* and *Q* are probability distributions then $\sum_{i=1}^{n}p_{i}=\sum_{i=1}^{n}q_{i}=1$ and the linear terms fall away giving the (normalized) KL-divergence (Eq 3). The Bregman divergences [16] are another important class of divergences between non-negative vectors, defined in terms of a strictly convex function, of which the generalized KL-divergence (Eq 6) is also a special case. Eq (6) is the definition that will be used throughout this paper, and is consistent with use by others, such as those within the signal processing and optimization community (e.g., [17, 18]). Note that the two fundamental properties remain intact; namely,

(i) non-negativity: KL(*P*, *Q*) ≥ 0 with equality if and only if *P* = *Q*, and
(ii) asymmetry: KL(*P*, *Q*) ≠ KL(*Q*, *P*).

At its essence KL(*P*, *Q*) is a coding penalty associated with selecting *Q* to approximate *P*. This KL-divergence also satisfies the homogeneity property of a distance:

$$\begin{array}{ccc} \mathrm{KL}(aP,aQ) & = & aP\;\text{ln}\frac{aP}{aQ}+aQ-aP,\\ & = & a\mathrm{KL}\left(P,Q\right),\;\text{for}\;\;\text{some}\;\;a\in R_{+}. \end{array}$$

It is informative to contrast the KL-divergence (Eq (6)) with the Euclidean distance, d, in a simple case: d(1001, 1000) = d(2, 1) = 1 whereas KL(1001, 1000) = 0.005 and KL(2, 1) = 0.3863. The discrepancy in KL values actually has greater similarity to that of the relative Euclidian distance (1/1001 ≈ 0.001 and 1/2 = 0.5). In this sense the Euclidian distance can be loosely thought of in terms of an absolute difference and the KL-divergence in terms of a relative difference.

The KL-divergence, as an information measure rather than a distance measure, will lead to an interesting new approach to data assimilation. Furthermore, the naturally embedded positivity constraint will prove useful to many problems applicable to data assimilation. Constraints are often required, or are desirable, to enforce non-negatively of certain physical quantities; such as length, volume and mass, variables such as precipitation and humidity, and concentrations of tracers. However, Kalman filter type approaches do not naturally handle such constraints. Quick-fix approaches such as simply setting negative values to zero are not optimal. Whereas the more involved approach of Gaussian anamorphosis (e.g. [19, 20]), whereby a nonlinear change of variables, such as a log transform, is introduced during the analysis step, are not ideal and may not be suitable for certain applications (e.g. [21]). More sophisticated constrained Kalman filtering data assimilation methods such as [22, 23] incorporate an optimization step that involves a projection into the constrained region. In contrast, our proposed KL-divergence filtering method will guarantee a positive state vector by construction; without need for any projection, transformations, ad-hoc adjustments, or any additional steps.

## Methods

In this section we review the traditional formulation of the data assimilation problem and outline sequential solution approaches. We then extend this formulation to describe a new data assimilation approach that utilizes Kullback-Leibler divergence.

### The data assimilation problem

Suppose at some time *t*_*k*_ we have partial observations, *y*_*k*_, and a background estimate, $x_{k}^{b}$, of some true state, $x_{k}^{t}$. The best estimate of that state, utilizing both background and observations, is given by the minimum, with respect to *x*, of an objective function, known as the 3D-Var cost function,

$$\begin{array}{c} J\left(x\right)=\frac{1}{2}\left|\right|H_{k}x-y_{k}{\left|\right|}_{R_{k}^{-1}}^{2}+\frac{1}{2}||x-x_{k}^{b}{\left|\right|}_{B_{k}^{-1}}^{2}. \end{array}$$ (7)

Here $\left|\right|\cdot {\left|\right|}_{A}^{2}={\langle \cdot ,\cdot \rangle }_{A}$ is a squared L_2_-norm weighted by a covariance matrix *A*, with the weighted inner product defined as 〈*a*, *b*〉_*A*_ = *a*^⊤^ *Ab*. The observations contain serially uncorrelated Gaussian errors, *μ*_*k*_ and are related to the state by an observation operator *H*_*k*_, such that $y_{k}=H_{k}x_{k}^{t}+\mu_{k}$ with $E(\mu_{k})=0$ and $E\left(\mu_{k}\mu_{k}^{⊤}\right)=R_{k}$. The background estimate also contains serially uncorrelated Gaussian errors, such that $E(x_{k}^{t}-x_{k}^{b})=0$ and $E\left(\left(x_{k}^{t}-x_{k}^{b}\right){\left(x_{k}^{t}-x_{k}^{b}\right)}^{⊤}\right)=B_{k}$. The minimum of the cost function (Eq 7) is a standard result given as:

$$\begin{array}{c} x_{k}=x_{k}^{b}+B_{k}H_{k}^{⊤}{\left(H_{k}B_{k}H_{k}^{⊤}+R_{k}\right)}^{-1}\left(y_{k}-H_{k}x_{k}^{b}\right). \end{array}$$ (8)

From a Bayesian perspective *x*_*k*_ is the expectation of the state of the system conditioned on the data, $x_{k}^{b}$ and *y*_*k*_ (see, for example, [7]). In the case of non-Gaussian errors this will still be the best linear unbiased estimator.

### The Kalman filter

In a sequentially updated system the background state is provided by the model forecast, $x_{k}^{f}$, at time *t*_*k*_, and is then updated by the best estimate (Eq 8) to give the analysis state, $x_{k}^{a}$, at time *t*_*k*_. This analysis state is then evolved forward in time by a numerical model of the evolving system, *M*_*k*,*k*+1_, to give the forecast state $x_{k+1}^{f}$, at time *t*_*k*+1_. In a similar manner the background errors are also evolved in time and updated to give a covariance forecast error, $P_{k+1}^{f}$, and a covariance analysis error, $P_{k+1}^{a}$, at time *t*_*k*+1_. For a linear system, with matrices *M*_*k*−1,*k*_ and *H*_*k*_, this process gives rise to the well-known Kalman Filter:

$$\begin{array}{cc} \text{forecast}\; & \left\{ \begin{array}{c} \;x_{k}^{f}=M_{k-1,k}\;x_{k-1}^{a},\\ \;P_{k}^{f}=M_{k-1,k}P_{k-1}^{a}M_{k-1,k}^{⊤}+Q_{k}, \end{array}\right. \end{array}$$ (9)

$$\begin{array}{cc} \text{analysis}\; & \left\{ \begin{array}{c} \;x_{k}^{a}=x_{k}^{f}+K_{k}\left(y_{k}-H_{k}x_{k}^{f}\right),\\ \;P_{k}^{a}=\left(I-K_{k}H_{k}\right)P_{k}^{f}, \end{array}\right. \end{array}$$ (10)

where

$$\begin{array}{c} K_{k}=P_{k}^{f}H_{k}^{⊤}{\left(H_{k}P_{k}^{f}H_{k}^{⊤}+R_{k}\right)}^{-1} \end{array}$$ (11)

is referred to as the Kalman gain matrix and *Q*_*k*_ denotes the covariance of the model error (assumed normally distributed, unbiased and serially uncorrelated).

### Kalman filter approximations

It is computationally expensive to propagate the error covariance matrix forward in time (Eq 9), prohibitively so for large systems. Furthermore, computing the full Kalman gain matrix (Eq 11), at each step, is typically impractical as it involves multiplying and inverting large matrices (which may be ill conditioned). As such, an approximation often used is to fix the error covariance matrix, that is, let *P*_*k*_ = *P*_0_ for all *t*_*k*_. This implementation method is often referred to as Optimal Interpolation (OI). To aid the inversion step the matrix is typically modified to have a simplified structure. For a non-linear model and/or observation operator a linearization about the background state is required in order to propagate covariances and this formulation is known as the extended Kalman filter (EKF). The ensemble Kalman filter (EnKF) is a popular implementation approach as it does not require a linear approximation, but instead involves propagating an ensemble of analysis vectors and then updating the ensemble using the observations, where the state vector is the ensemble mean and the state error covariance matrix is constructed by the ensemble covariance matrix (see, for example, [24]).

### Kullback-Leibler regularization

The use of Kullback-Leibler minimization for static inverse problems has previously been established. For example, Resmerita and Anderssen [25] have highlighted the choice of KL-divergence as both residue minimizer and regularizer in a two-term cost function for solving ill-posed linear inverse problems. We now describe two iterative methods for minimizing functionals involving additive KL-divergence terms.

#### Expectation maximization (EM)

The expectation maximization (EM) algorithm [17] was originally used by Byrne [26] to determine the solution to the following regularization problem:

For $x\in R_{+}^{N}$ and 0 ≤ *α* ≤ 1 minimise

$$\begin{array}{c} J(x)=\alpha \mathrm{KL}(d,Tx)+(1-\alpha )\mathrm{KL}(q,x), \end{array}$$ (12)

to solve a possibly inconsistent linear system *Tx* = *d*, where *T* ∈ R^*M*×*N*^ and $d\in R^{M}$, and where $q\in R^{N}$ is an a priori estimate of *x*.

The solution, equivalent to maximizing Burg entropy, is derived from alternating minimization of related KL distances between convex sets [26]. Following Qranfal and Byrne [27] the iterative solution can be described in a single step:

$$\begin{array}{c} x_{j}^{\ell +1}=\alpha x_{j}^{\ell }\sum_{i=1}^{M}\frac{T_{ij}d_{i}}{{\left(Tx^{\ell }\right)}_{i}}+\left(1-\alpha \right)q_{j}, \end{array}$$ (13)

where for some initial start vector *x*^0^ > 0, *ℓ* is iterated until convergence, provided that $\sum_{i=1}^{M}T_{ij}=1$ for each *j*.

#### The simultaneous multiplicative algebraic reconstruction technique (SMART)

The simultaneous multiplicative algebraic reconstruction technique (SMART) was introduced by Bryne [17, 26] as a means of solving the following problem:

For *x* > 0 and 0 ≤ *α* ≤ 1 minimise

$$\begin{array}{c} J(x)=\alpha \mathrm{KL}(Tx,d)+(1-\alpha )\mathrm{KL}(x,q), \end{array}$$ (14)

to approximate the solution to the linear system of equations *Tx* = *d* with *q* an a priori estimate of *x*. Recall that the KL-divergence is not symmetric, hence Eq (14) is formally a different problem from that of Eq (12).

The solution to Eq (14), equivalent to maximizing Shannon entropy, was determined by Byrne [26] using a two-step alternating projections algorithm, which can be expressed in a convex combination compact form [28]:

$$\begin{array}{c} x_{j}^{\ell +1}={\left(q_{j}\right)}^{1-\alpha }[x_{j}^{\ell }\prod_{i=1}^{M}(\frac{d_{i}}{{\left(Tx^{\ell }\right)}_{i}}{)}^{T_{ij}}{]}^{\alpha }, \end{array}$$ (15)

where *ℓ* is iterated until convergence, starting from an initial guess *x*^0^ > 0.

### Kullback-Leibler based data assimilation

The cost functions of Eqs (12) and (14) can be reformulated to solve a data assimilation problem analogous to Eq (7). In this context the linear system *Tx* = *d* can be used to represent how the observations *y* are related to the state vector *x* through the observation operator *H*, namely *Hx* = *y*, and the a priori estimate *q* is given by the model forecast *x*^f^. For example, taking the case from Eq (12), we can derive a weighted Kullback-Leibler objective function, using our data assimilation notation, as follows:

$$\begin{array}{c} J\left(x\right)={\mathrm{KL}}_{R_{k}^{-1}}\left(y_{k},H_{k}x\right)+{\mathrm{KL}}_{{\left(P_{k}^{f}\right)}^{-1}}\left(x_{k}^{f},x\right). \end{array}$$ (16)

The covariance matrices, *R*_*k*_ and $P_{k}^{f}$, and their inverses, are symmetric positive definite and by the Cholesky decomposition may be expressed in the form:

$$R_{k}^{-1}=U_{k}^{⊤}U_{k},$$ (17)

$${\left(P_{k}^{f}\right)}^{-1}=V_{k}^{⊤}V_{k},$$ (18)

where *U* and *V* are upper triangular matrices with positive diagonal entries. Just as, $\left|\right|H_{k}x-y_{k}{\left|\right|}_{R_{k}^{-1}}^{2}=\left|\right|U_{k}\left(H_{k}x-y_{k}\right){\left|\right|}^{2}$, for example, so to for weighted KL-divergence, hence

$$\begin{array}{c} J\left(x\right)=\mathrm{KL}\left(U_{k}y_{k},U_{k}H_{k}x\right)+\mathrm{KL}\left(V_{k}x_{k}^{f},V_{k}x\right). \end{array}$$ (19)

The KL-divergence must be between two positive quantities (see Eq 6); therefore, we are restricted in that we require positive entries for *U*_*k*_ *y*_*k*_, *U*_*k*_ *H*_*k*_ *x*, $V_{k}x_{k}^{f}$, and *V*_*k*_ *x*. Suppose we have a positive system, such that *y*_*k*_, *H*_*k*_, and $x_{k}^{f}$ each have positive entries. This would be the case for a wide range of applied science applications, and if not a general system can always be converted to a positive system after applying some transformations (see, for example, [28]). However, even with the supposed positive system, *U*_*k*_ and *V*_*k*_ may still have negative off-diagonal entries. For this derivation we will therefore restrict ourselves to white noise for the observation error and the forecast error, such that

$$R_{k}={\left(\sigma_{o}\right)}_{k}^{2}I,$$ (20)

$$P_{k}^{f}={\left(\sigma_{k}^{f}\right)}^{2}I.$$ (21)

This is the usual structure for the observation error (Eq 20) as observations are typically local in space and considered independent; however, for the forecast error (Eq 21) this form does present some restrictions as it limits our ability to spread information from an observed part of the system to an unobserved part. We will circumvent this to some extent by interpolating observations to all grid points and assimilating these interpolated values, with reduced weight the further they are from the measurement location. This localization procedure enables the smooth spatial spread of information from the measurement point to nearby locations, without the need for off-diagonal terms in the covariance matrix.

Proceeding with (20) and (21) we have $U_{k}=\frac{1}{{\left(\sigma_{o}\right)}_{k}}I$ and $V_{k}=\frac{1}{{\left(\sigma^{f}\right)}_{k}}I$, with (*σ*_o_)_*k*_, (*σ*^f^)_*k*_ > 0 for all *k*, and after some algebraic adjustments we can derive

$$\begin{array}{c} \hat{J}\left(x\right)=\frac{\sigma_{k}^{f}}{\sigma_{k}^{f}+{\left(\sigma_{o}\right)}_{k}}\mathrm{KL}\left(y_{k},H_{k}x\right)+\frac{{\left(\sigma_{o}\right)}_{k}}{\sigma_{k}^{f}+{\left(\sigma_{o}\right)}_{k}}\mathrm{KL}\left(x_{k}^{f},x\right). \end{array}$$ (22)

We now have a weighted KL objective function that matches the form of Eq (12) and the minimum can be found using the iterative method of Eq (13). This can be solved sequentially as a filtering algorithm by evolving the model state and updating the forecast based on the observations. We have therefore outlined an EM data assimilation filter that can be compared to traditional 3D-Var/OI. Namely,

$$\begin{array}{c} \begin{array}{cc} x_{k}^{f} & =M_{k-1,k}\;x_{k-1}^{a}\\ (x_{k}^{a}{)}_{j}^{\ell +1} & =\alpha_{k}(x_{k}^{a}{)}_{j}^{\ell }\sum_{i=1}^{M}\frac{{\left(H_{k}\right)}_{ij}{\left(y_{k}\right)}_{i}}{{\left(H_{k}{\left(x_{k}^{a}\right)}^{\ell }\right)}_{i}}+\left(1-\alpha_{k}\right)(x_{k}^{f}{)}_{j} \end{array} \end{array}$$ (23)

for *j* = 1, …, *N* and *ℓ* is iterated until convergence, and where $\alpha_{k}=\frac{\sigma_{k}^{f}}{\sigma_{k}^{f}+{\left(\sigma_{o}\right)}_{k}}$.

A similar procedure can be followed to produce a SMART data assimilation filter

$$\begin{array}{c} \begin{array}{cc} x_{k+1}^{f} & =M_{k-1,k}\;x_{k-1}^{a}\\ (x_{k}^{a}{)}_{j}^{\ell +1} & =(x_{k}^{f}{)}_{j}^{1-\alpha_{k}}[(x_{k}^{a}{)}_{j}^{\ell }\prod_{i=1}^{M}(\frac{{\left(y_{k}\right)}_{i}}{{\left(H_{k}{\left(x_{k}^{a}\right)}^{\ell }\right)}_{i}}{)}^{{\left(H_{k}\right)}_{ij}}{]}^{\alpha_{k}} \end{array} \end{array}$$ (24)

for *j* = 1, …, *N* and *ℓ* is iterated until convergence, and where $\alpha_{k}=\frac{\sigma_{k}^{f}}{\sigma_{k}^{f}+{\left(\sigma_{o}\right)}_{k}}$.

Hence, we have now developed two new data assimilation methods that minimize Kullback-Leibler divergence. What we have proposed is an entirely new perspective to the traditional data assimilation scheme, one that involves an information measure for the closeness of fit between model and data. Furthermore, these KL data assimilation algorithms guarantee positivity for the solution without the need for projections or transformations and they do not require an adjoint code or the storage, multiplication or inversion of covariance matrices.

## Numerical experiments

We examine the performance of the EM and SMART data assimilation filters with respect to Optimal Interpolation (OI) and the Kalman filter (KF), including the extended Kalman filter (EKF) and the ensemble Kalman filter (EnKF) for a non-linear application. To demonstrate and compare these algorithms we perform so-called ‘twin experiments’ whereby noisy pseudo-observations, $y_{k}=H_{k}x_{k}^{t}+\mu_{k}$, are taken from a truth run $x_{k+1}^{t}=M_{k,k+1}x_{k}^{t}+\epsilon_{k}$. The initial model state is offset from the truth, $\delta =x_{0}^{t}-x_{0}^{a}$, the model is evolved forward in time, $x_{k+1}^{f}=M_{k,k+1}x_{k}^{a}$, and the observations are assimilated in an attempt to recover the truth run. The error statistics are such that

$$E(\mu_{k})=0\;E\left(\mu_{k}\mu_{k}^{⊤}\right)=R=\sigma_{o}^{2}I,$$ (25)

$$E(\epsilon_{k})=0\;E\left(\epsilon_{k}\epsilon_{k}^{⊤}\right)=Q=\sigma_{m}^{2}I,$$ (26)

$$E(\delta )=0\;\text{Var}\left(\delta \right)=\sigma_{b}^{2}\;E\left(\delta \delta^{⊤}\right)=P_{0}^{a}.$$ (27)

We will demonstrate the KL-minimizing data assimilation methods (EM filter and SMART filter) using three different numerical experiments.

### Experiment 1

**Fig 1:**
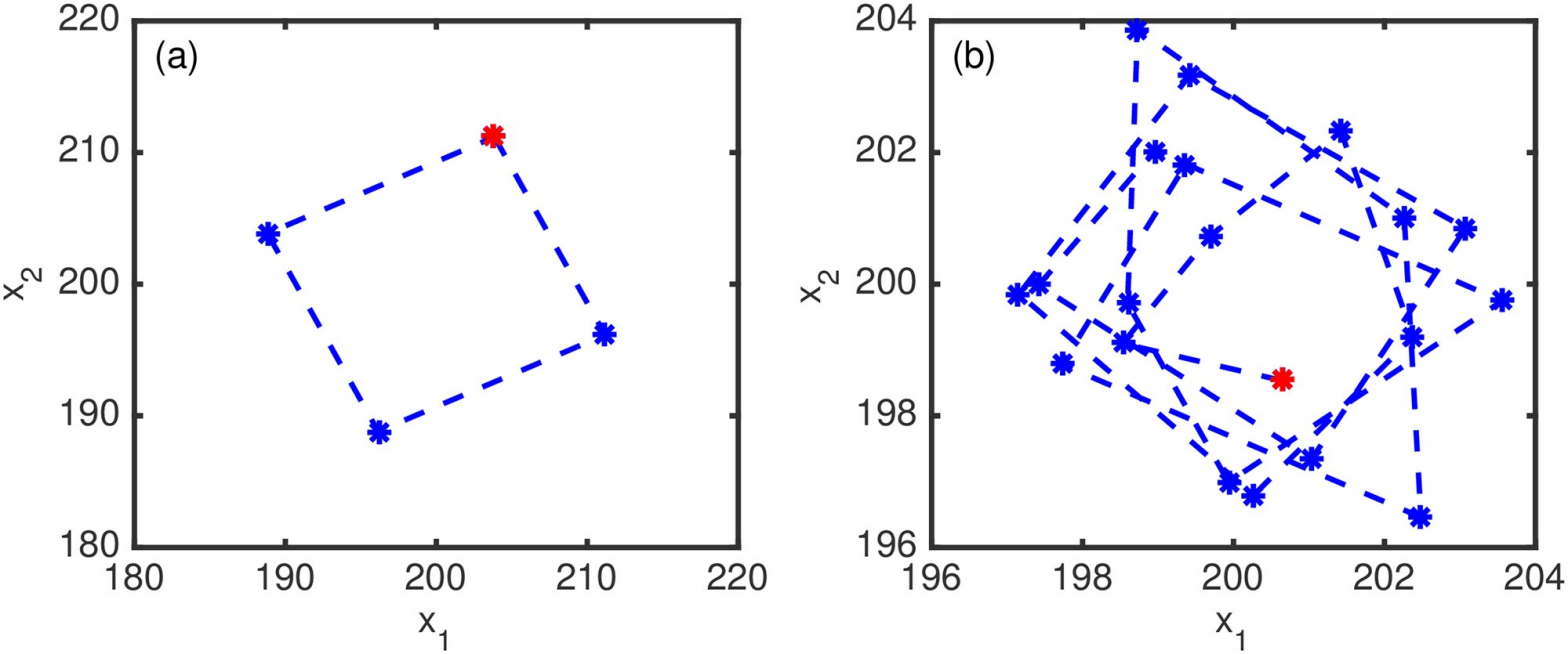
Experiment 1. The modelled phase plane of the background solution (a) and the truth solution (b). The initial conditions are given by the red dot and the solution points are in blue.

We first consider a two-dimensional linear dynamics problem taken from [8]

$$\begin{array}{c} {}[\begin{array}{c} x_{1}\\ x_{2} \end{array}{]}_{k+1}=[\begin{array}{cc} 0 & 1\\ -1 & 0 \end{array}][\begin{array}{c} x_{1}\\ x_{2} \end{array}{]}_{k} \end{array}$$ (28)

in which the state vector is rotated 90° in a clockwise direction at each step. To perform these simulations in the positive quadrant we rotate about the point $[\begin{array}{cc} 200 & 200 \end{array}{]}^{⊤}$. We first translate the state vector so that the point of translation is moved to the origin, then we rotate the relocated state vector about the origin (Eq 28), finally we undo the translation step to return the state vector to its new rotated location. Within our twin-experiment the background guess follows these deterministic dynamics; however, the truth solution involves the addition of random noise to Eq 28, producing stochastic dynamics as the noise causes a random shift of the origin (see Fig 1). The experiments are performed with an initial condition offset from $[\begin{array}{cc} 200 & 200 \end{array}{]}^{⊤}$, where the offset is taken from the normal distribution with mean 0 and variance $\sigma_{b}^{2}=10$. The random model error (that produces the stochastic dynamics) is normally distribution with mean zero and variance $\sigma_{m}^{2}=1$. Observations are generated from the truth run at each step from the *x*_1_ variable only and include a normally distributed random measurement error of mean zero and variance $\sigma_{o}^{2}=1$.

### Experiment 2

**Fig 2:**
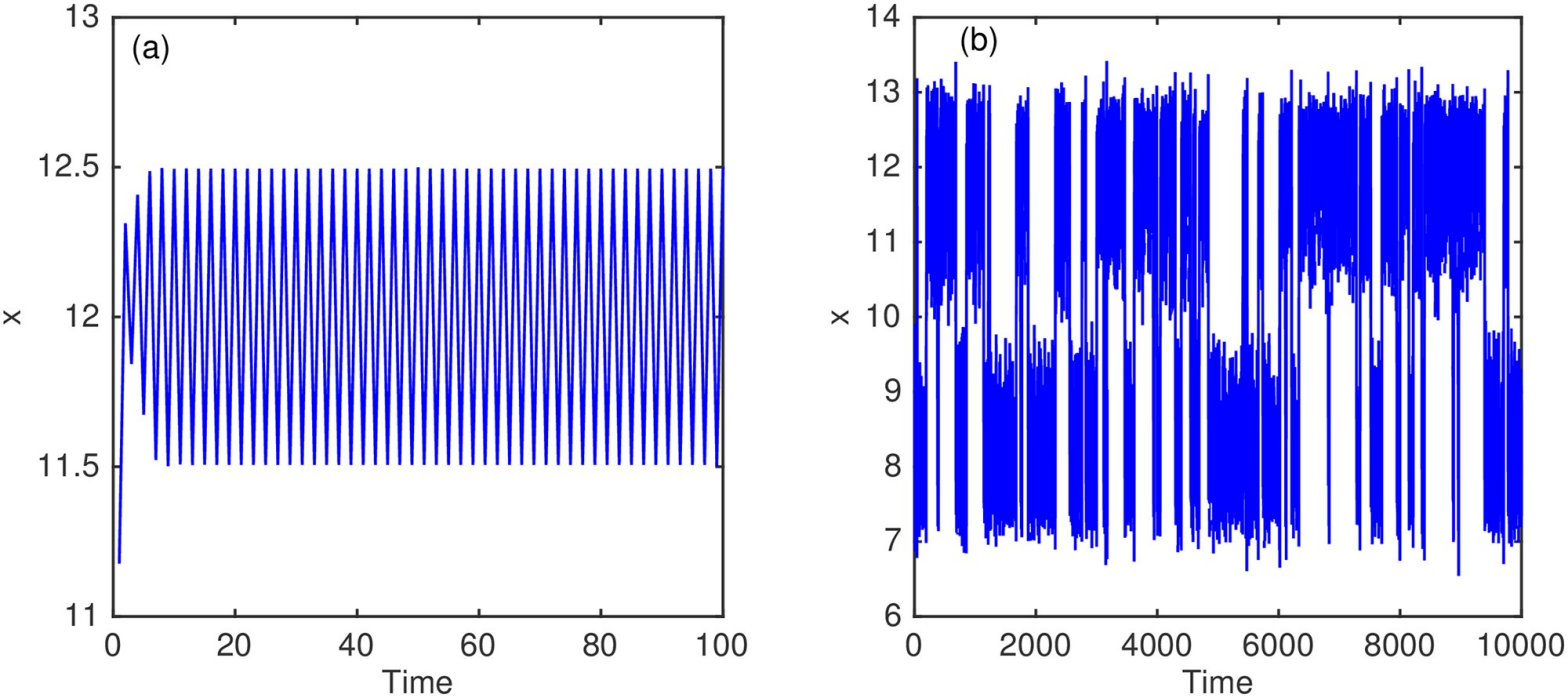
Experiment 2. The modelled background solution (a) and the truth solution (b).

This one-dimensional non-linear dynamics problem involves a sine map

$$\begin{array}{c} x_{k+1}=\frac{5}{2}\text{sin}\left(x_{k}\right) \end{array}$$ (29)

and produces deterministic behaviour converging to a period-2 solution; however, the addition of noise creates stochastic dynamics producing considerably different bistable behaviour between two separate period-2 solutions [8]. We start with a mean initial condition of 10. To run this experiment with positive values we subtract 10 from the solution before applying the forward model (Eq 29) and then add 10 after the sine map has been applied (see Fig 2). The stochastic behaviour of the truth run is generated with the addition of normally distributed model errors of mean 0 and variance $\sigma_{m}^{2}=0.09$. The model forecast has an initial condition error that is taken from the normal distribution with mean 0 and variance $\sigma_{b}^{2}=0.9$. Observations are acquired from the truth run at each step and measurement error is added that is normally distributed with mean 0 and variance $\sigma_{o}^{2}=1$.

### Experiment 3

In this example we have a spatio-temporal model, a partial differential equation with a single spatial variable, *χ*, namely the one-dimensional linear advection equation,

$$\begin{array}{c} \frac{\partial \chi }{\partial t}+v\frac{\partial \chi }{\partial x}=0. \end{array}$$ (30)

Here the state vector is the 1-D concentration *χ* = *χ*(*x*, *t*) which is advected with constant fluid velocity *v*. This model (Eq 30) has an analytical solution, which we will use in this study,

$$\begin{array}{c} \chi (x,t)=\chi (x-vt,0). \end{array}$$ (31)

We take *v* = 1 m s^−1^, discretize with spacing 1 m and use a timestep of 1 s. For the baseline experiments we have 400 spatial gridpoints and evolve over 600 timesteps. We apply periodic boundary conditions. The state vector, *χ*, is initialized as a pseudo-random wave (Fig 5(a)). This smooth periodic initial state is sampled from a normal distribution with mean 0, variance $\sigma_{b}^{2}=5$, and a decorrelation length of 20. The solution consists of a superposition of sinusoids with different wavelengths, where the shorter waves are penalized, and where each wave has a random phase [24]. The background state at initialization is offset from the truth by drawing another sample from the distribution and adding this to the true state. Observations are taken from the truth run and used in the assimilation (see Fig 5(b), 5(c)). Every 12 timesteps 20 observations are taken from the 400 possible spatial locations which are randomly sampled from a uniform distribution without replacement. Observation error is added to each observation, where the noise is normally distributed with mean zero and variance $\sigma_{o}^{2}=0.05$.

## Results and discussion

For the experiments conducted in this work we found that the two KL-minimizing data assimilation methods provided near-identical solutions, we will therefore only present the results from the EM filter which we will henceforth refer to as the Kullback-Leibler data assimilation (KL-DA) solution. To give a sense of how differences might arise consider Eq 1, when minimizing KL(*P*, *Q*) we want *P* ≃ *Q* or *P* ≪ *Q*, now suppose *Q* has two peaks then *P* might match one peak (*P* ≃ *Q*) and miss the other (*P* ≪ *Q*), we might think of this as “mode-seeking”, whereas for minimizing KL(*Q*, *P*) we want *P* ≃ *Q* or *Q* ≪ *P*, hence *P* might allocate mass between the two peaks of *Q*, thus “mean-seeking” (see [29]). Different applications might then give rise to different solutions from the EM filter and the SMART filter, although this is not something we have explored in this study.

**Fig 3:**
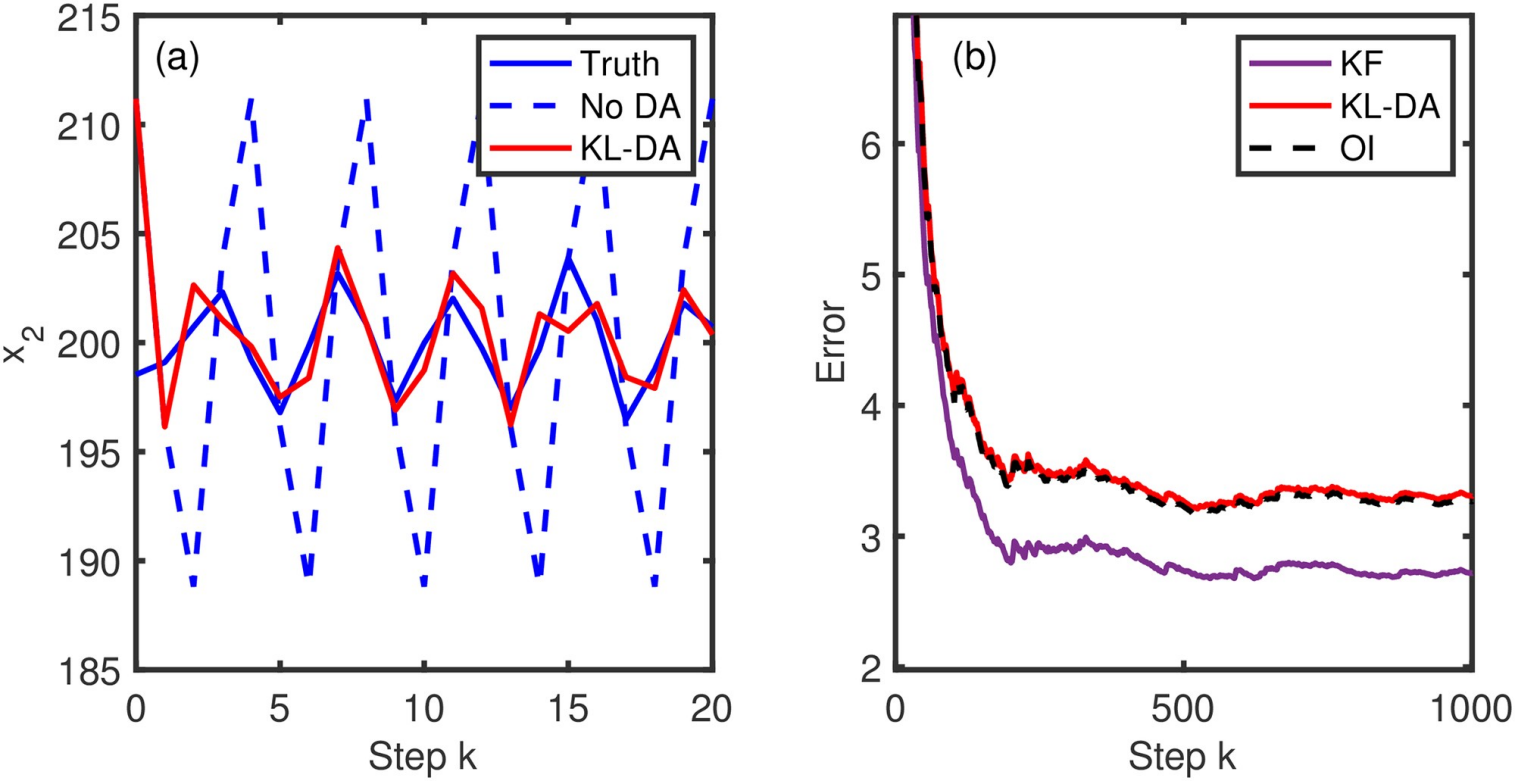
Experiment 1 results. The modelled solution (a) and running average root mean square error (b).

In experiment 1 we show that the KL-DA method is effective and accurately tracks the unobserved variable (Fig 3(a)). The KL-DA results are shown to be equivalent to the OI solution, with the Kalman filter solution being superior to both (Fig 3(b)). This is to be expected as the full Kalman filter is updating the error covariances through the simulation, unlike the static covariance of the OI and KL-DA systems.

**Fig 4:**
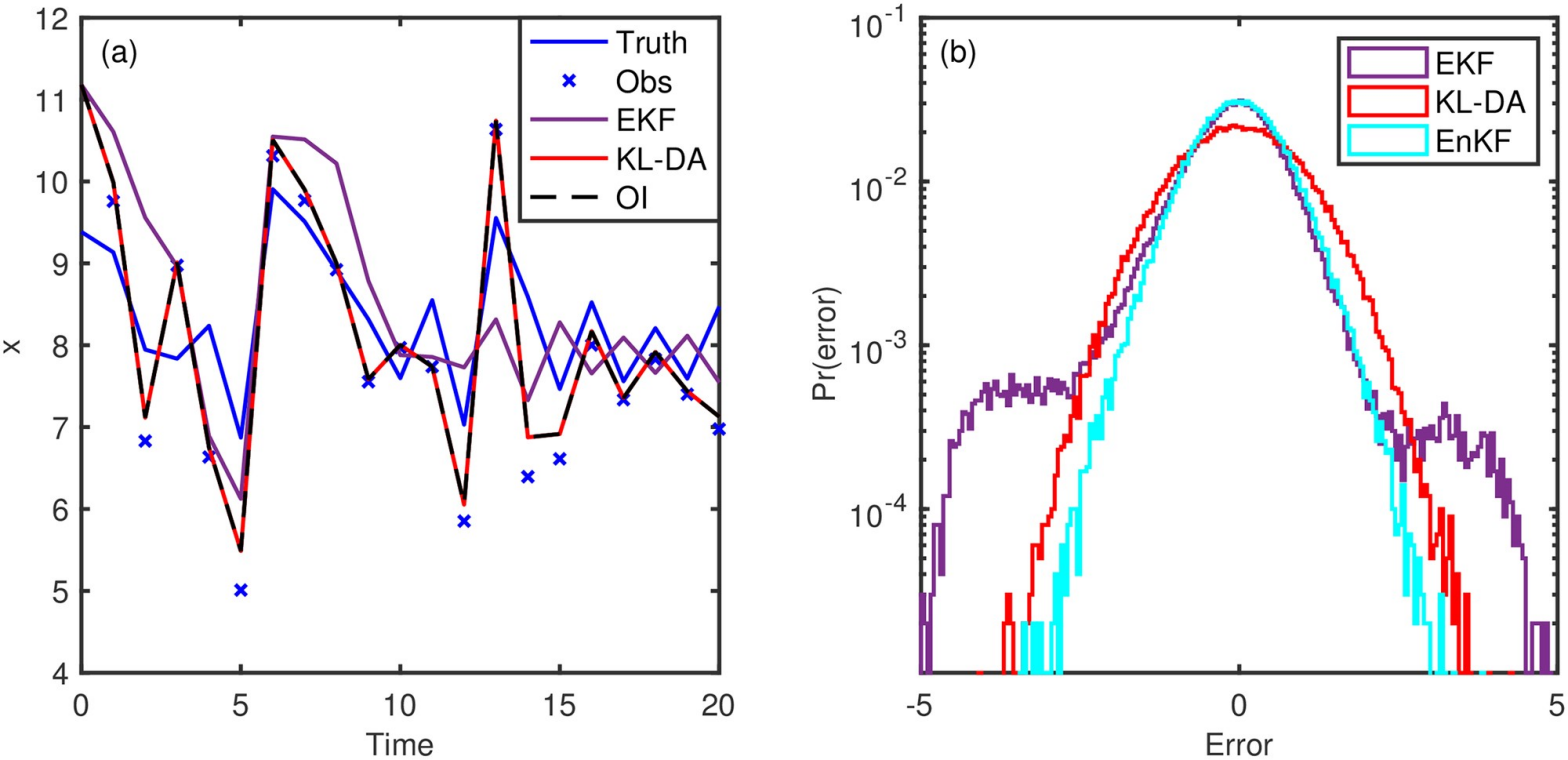
Experiment 2 results. The modelled data assimilation solutions (a) and the log-scale histogram of the errors (b).

For the 1-D nonlinear problem, experiment 2, we again find that the solution of the KL-DA method is identical to that of the OI (Fig 4(a)). We find that the KL-DA (and OI) is more accurate than the extended Kalman filter, which produces a higher frequency of larger errors, as shown in the probability histrogram of the errors (Fig 4(b)). This problem is not well suited for the extended Kalman filter because of destablization intervals [8]. The ensemble Kalman filter (EnKF) solution is found to have a slightly narrower range of error values than the KL-DA and OI (Fig 4(b)), but this comes from considerably greater computational cost (here we used 100 ensemble members to characterize the evolving error statistics).

**Fig 5:**
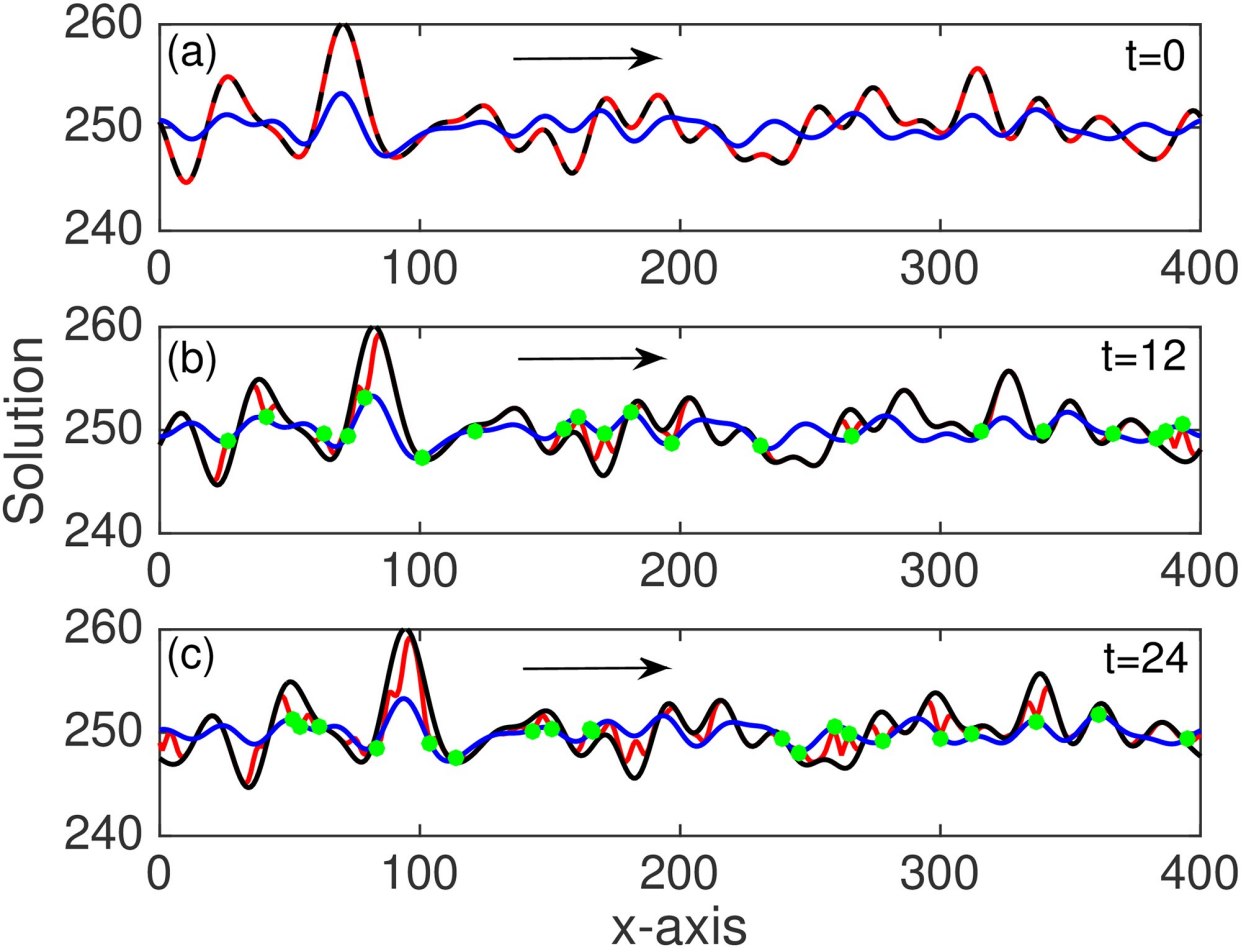
Experiment 3. The state vector solution from the truth run (blue), the no assimilation run (black), and the Kullback-Leibler data assimilation solution (red). Shown at (a) the initial condition (t = 0), (b) the time of first observations (t = 12), and (c) the time of second observations (t = 24). The observations (green circles) are taken at random locations and include random measurement error. The arrow indicates the direction of the advected flow.

**Fig 6:**
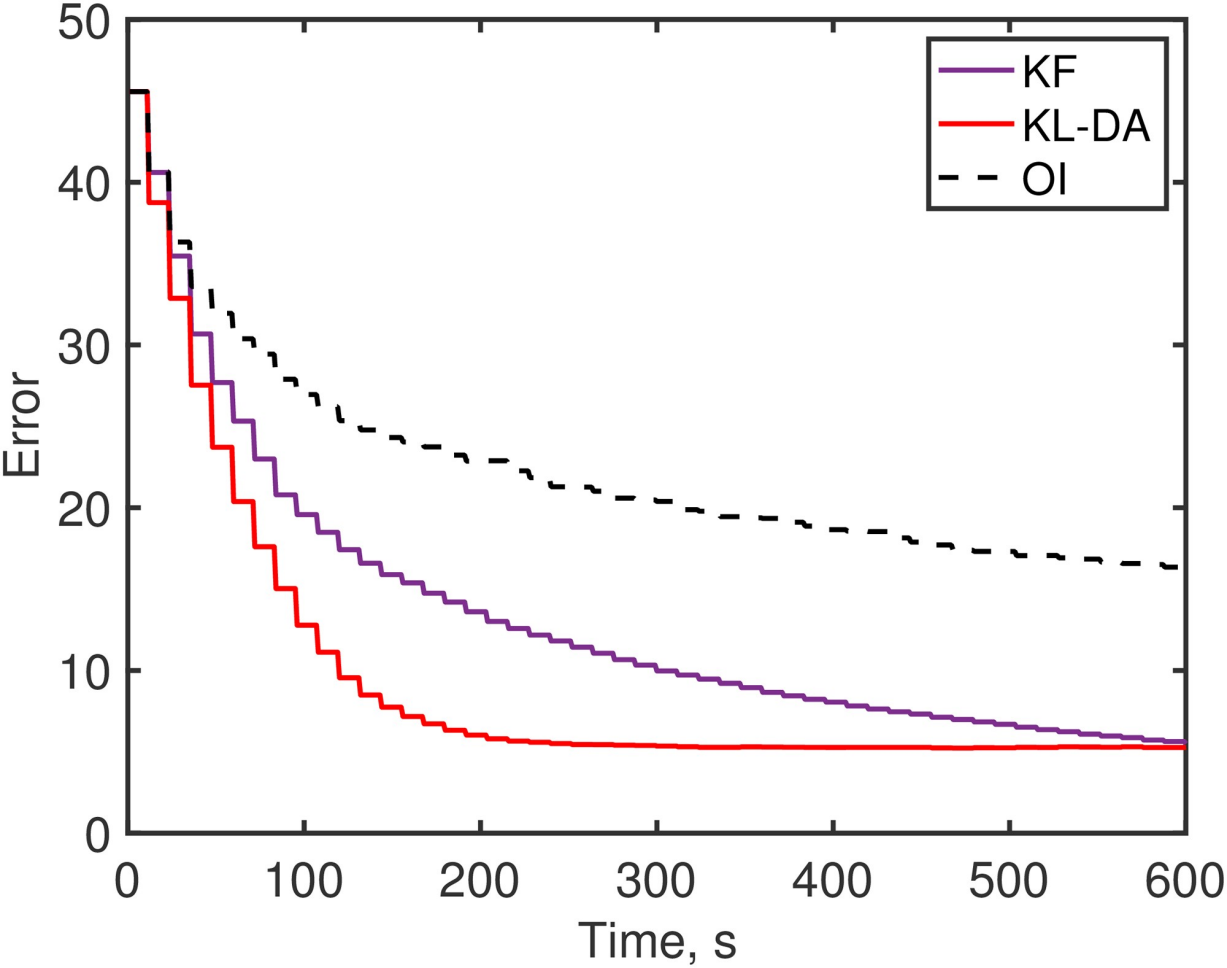
Experiment 3 error. The error, $\left|\right|\chi^{t}-\chi {\left|\right|}_{2}^{2}$, growth over time using the Kalman filter (purple), the Kullback-Leibler data assimilation (red), and the OI (black dashes) solutions. The error curves are determined based on the mean of 100 simulations.

In experiment 3 we have background error with a decorrelation length scale and so the error covariance matrix, *P*^f^, contains important off-diagonal structure (unlike in experiments 1 and 2). As the KL-DA method uses diagonal covariance matrices we employ a local assimilation approach, as detailed in our earlier derivation. The observations are linearly interpolated and assimilated at each grid point. The uncertainty assigned to these interpolated observations is increased exponentially the further they are from the actual observation location, such that beyond a certain distance the uncertainty is so great that the interpolated observation will have no bearing on the analysis. This is an effective way of spatially spreading the observation information to locations nearby the measurement points (see Fig 5(b) and 5(c)). It is also different from that of OI (and Kalman filter) which use the *P*^f^ matrix in the Kalman gain to spread information; hence, the KL-DA and OI solutions are no longer alike as was the case for the previous experiments. We find that the KL-DA solution converges toward the truth much faster than both OI and the Kalman filter (Fig 6). As expected errors are reduced much more slowly in the OI than the Kalman filter as the error covariance is not evolved or updated (Fig 6). Note that as each individual simulation will be different, because of random errors and the random selection of observation locations, we have presented our results (in Fig 6 and Table 1) as averages calculated from multiple realizations (repeat simulations).

**Table 1:** The average time to complete the simulation and the average error $\left|\right|\chi^{t}-\chi {\left|\right|}_{2}^{2}$ at the end of the simulation *t* = 600 depending on the size of the domain and the number of observations assimilated every 12 timesteps. These averages are mean values taken over 30 simulations using experiment 3.

Grid size	No. Obs.	KF		KL-DA		OI	
		Time (s)	Error	Time (s)	Error	Time (s)	Error
200	10	3.70	4.65	1.04	6.83	0.52	16.73
400	20	9.70	5.49	2.14	5.29	1.38	17.55
800	40	48.60	3.82	7.80	4.86	6.94	8.58
1600	80	324.1	5.46	20.54	6.10	20.92	12.08
3200	160	2396	7.65	66.30	8.22	80.14	16.47

The KL-minimizing data assimilation methods are found to be substantially faster than the Kalman filter and faster than OI for large systems (see Table 1). The algorithms have not necessarily been coded for optimal efficiency; nonetheless, these timing comparisons provide further evidence of the computational advantages of the KL data assimilation approach, especially for large systems. The efficiency of the EM and SMART filters partly derives from its avoidance of matrix algebra. In contrast the Kalman filter approaches depend critically on the Kalman gain matrix (Eq 11), determining this requires computing the inverse of the matrix (*HP*^f^ *H*^⊤^ + R). Even if *P*^f^ is fixed, as is the case for OI, the *H* matrix (observation operator) will be different due to the changing measurement locations in experiment 3. Thus requiring a new inverse to be computed at each assimilation time. The challenges of matrix multiplication, matrix storage, and computing matrix inverses increase substantially with the size of the system and may become intractable for some very large applications. For example, as we increase the number of grid points in our study we find the time needed to complete the simulations dramatically increases for the Kalman filter (see Table 1). As is well known evolving and updating the error covariance in the Kalman filter requires significant additional computational resources over that of OI. Although in practice the EnKF proves effective as it does not propagate a covariance matrix, but instead makes an ensemble forecast. For high dimensional applications the EnKF can be implemented by assimilating single observations serially (e.g. [30]) or by performing the analysis step in a local region (e.g. [31]). Regardless, any ensemble forecast will be more expensive than the single forecast of the KL-DA method; nonetheless it could be worth pursuing some of the benefits of the EnKF by developing an ensemble approach to the KL-DA method.

With regards to accuracy, we find that the final solutions of the KF are much closer to the truth than the OI solutions, but only slightly better than the KL-DA method (see Table 1). For example, for the largest system tested the relative percentage error at the end of the simulation for KF was 3.06%, for KL-DA was 3.29% and for OI was 6.59%. Whereas, the KL-minimizing filter is substantially quicker than the Kalman filter and is found to be also faster than the OI (direct solve 3D-Var) for sufficiently large systems (see Table 1). For example, in the largest problem tested the KL-DA simulation was 36 times faster to complete than the KF and 1.2 times faster than the OI. Therefore, the larger the system the more advantageous the KL data assimilation approaches become. These comparisons provide a baseline for assessing the KL-method and give an indication of their potential.

For all experiments performed in this study only a couple of iterations of the SMART and EM filters are needed for convergence |*x*^*ℓ*^ − *x*^*ℓ*−1^| < 10^−9^. We should emphasis that the OI solution involves computing the direct and exact solution (Eq 8), but that iterative 3D-Var methods can also be employed to find the cost function minimum (e.g., [32]); however, such numerical minimization algorithms require evaluating both the cost function as well as its gradient. As such we expect the computational advances of the KL-DA approach to remain against the iterative minimization algorithms of 3D-Var.

A shortcoming of the KL data assimilation set-up described is the assumed diagonal structure of the covariance matrix. Realistic multivariable data assimilation applications typically require at least tri-diagonal structure in order to adjust correlated variables. Future work will explore adaptions to the current KL-minimizing filters in order to address this limitation and increase their utility. For example, an ensemble KL-DA approach could be developed that allows for both covariance updating and the direct adjustment of unobserved, but correlated, variables.

Many applications in the applied sciences require constraints on state variables; for instance, negative quantities are not physically possible for sea-ice concentration or ice sheet thickness, to give a couple of examples. Nonetheless, even with positive observations and a positive forecast vector, the data assimilation update (Eq 10) can result in physically unsound negative values occurring in the analysis state. Despite a positive system the innovation vector *y* − *Hx*^f^ could be negative or the Kalman gain (Eq 11) could contain negative values because of the matrix inverse. Often, in such cases any negative values in the analysis state vector are simply set to zero in a post-processing step necessary to maintain consistency with the physical model (see, for example, [33, 34]). However, this decision is rather ad-hoc and is no longer the optimal solution provided by the data assimilation algorithm. Another frequently used approach is that of Gaussian anamorphosis and involves a change of variables for the state vector and the observations, the nonlinear transformation is applied before the update step and then the inverse is used to return back to physical space for the forecast (e.g. [19]). The application of anamorphosis functions for these transformations may not be straightforward and the choice can strongly influence performance (e.g. [20]), although the logarithm is a popular function choice. Despite these approaches giving non-negative analyses they are not necessarily optimal and do not generally conserve mass (e.g. [21]). To counteract such shortcomings more elaborate techniques have been developed that involve solving an optimization problem subject to convex constraints at the analysis step, see for example [21–23]. In contrast, the data assimilation methods we have formulated here are guaranteed to produce analysis states with positive values because they are based on KL-divergence and hence are ideally suited for any applications requiring such physical constraints. No adjustments, transformations, or projections are required and the constraint is naturally embedded in the KL-minimizing filtering algorithms. For example, in our twin experiment 3 problem if we produce the initial pseudo-random wave around the zero line and then offset by the minimum observation (to achieve a positive system) and run the data assimilation experiments with reduced observation error (e.g., $\sigma_{o}^{2}=0.01$) then the Kalman filter solution produces multiple (undesired) negative values. However, when using the KL-minimizing filters there are no occurrences of negative values and positivity is naturally enforced. Future work will build on this potential by exploring more realistic applications that require positivity and directly comparing the outcomes of KL-DA to Gaussian anamorphosis and other competing approaches.

The Kullback-Leibler divergence has previously been used within the standard data assimilation methods. For example, Mansouri et al. [35] minimize KL divergence to generate the optimal importance proposal distribution within a particle filter. This KL ‘measure’ is also used for model selection via Akaike information criterion, see for example, Burnham and Anderson [36]. In particular, Lang et al. [37] used such a method within an ensemble Kalman filter for parameterization estimation. The Kullback-Leibler divergence has recently been used to incorporate inequality constraints for an ensemble Kalman filter [23]. Their methodology involves first solving the unconstrained ensemble Kalman filter and then projecting these results into the constrained region. In the projection step they seek a distribution in the constrained region that is similar and close to that of the unconstrained region and to determine this they solve a convex optimization problem using the KL-divergence. In contrast our approach can be considered favourable in that we guarantee our solution, by construction and without projection, to belong to the desired constrained region, namely the positive octant for this study. We have originally demonstrated that data assimilation methods can be developed that seek to minimize the Kullback-Leibler divergence, between model forecast and observations as well as between the forecast and the control state, within a two-term weighted cost function. We have shown that these new approaches are computationally efficient and are ideally suited for situations where physical constraints on the state vector are necessary. Such scenarios commonly arise within many state and parameter estimation problems across numerous disciplines.

## Conclusion

We have derived two new data assimilation algorithms that minimize Kullback-Leibler divergence, rather than the L_2_-norm of standard data assimilation methods. This foundational information-based perspective provides a new way to conceptualize the data assimilation problem. The unnormalized Kullback-Leibler divergence is a measure of the discrepancy between two positive vectors, and is a natural way to characterize the differences between the model prediction and the data. Because this ‘measure’ is not symmetric we have developed two independent filtering schemes, namely the simultaneous multiplicative algebraic reconstruction technique (SMART) filter and the expectation maximization (EM) filter. These proposed KL data assimilation schemes have been implemented numerically and the results compared to Kalman filter approaches. The two algorithms (EM filter and SMART filter) are shown to provide near-identical solutions with accuracy matching the 3D-Var solution using the Optimal Interpolation (OI) method with the same information inputs. We have highlighted several advantages of the KL-based data assimilation methods and indicated the future potential of this approach. The KL methods are computationally much faster than the Kalman filter as they are iterative schemes that have no need for matrix storage, matrix multiplication or computing a matrix inverse. For larger systems the KL-based data assimilation approach is shown to have substantial computational advantages over the Kalman filter and 3D-Var/OI. Furthermore, the KL data assimilation methods are ideal for applications that require state variables (or parameters) to obey certain constraints, such as physical limitations on their values. The KL-divergence applies to positive vectors only and so naturally embeds a constraint without any need for additional steps, such as transformations or projections, unlike the Kalman filter schemes. We have outlined important theoretical and conceptual details and highlighted how this promising new approach can be further improved by focusing on adapting the methods so that error covariance can be evolved and more complicated covariance structure can be incorporated. The KL-DA framework developed in this paper will be used as a foundation for future work demonstrating the methods in more sophisticated applications. In summary, the proposed Kullback-Leibler minimizing filtering methods provide a new data assimilation framework that might hold potential for applications involving time-varying variables of large-scale systems and where physical constraints and limited computational resources present challenges.
